# The effects and significance of gut microbiota and its metabolites on the regulation of osteoarthritis: Close coordination of gut-bone axis

**DOI:** 10.3389/fnut.2022.1012087

**Published:** 2022-09-20

**Authors:** Lei Liu, Feng Tian, Guo-Yuan Li, Wei Xu, Rui Xia

**Affiliations:** Department of Orthopedics, The First Affiliated Hospital of USTC, Division of Life Sciences and Medicine, University of Science and Technology of China, Hefei, China

**Keywords:** osteoarthritis, gut microbiota, metabolites, gut-bone axis, bone

## Abstract

Osteoarthritis (OA) is a common chronic degenerative disease of articular cartilage in middle-aged and older individuals, which can result in the joint pain and dysfunction, and even cause the joint deformity or disability. With the enhancing process of global aging, OA has gradually become a major public health problem worldwide. Explaining pathogenesis of OA is critical for the development of new preventive and therapeutic interventions. In recent years, gut microbiota (GM) has been generally regarded as a “multifunctional organ,” which is closely relevant with a variety of immune, metabolic and inflammatory functions. Meanwhile, more and more human and animal researches have indicated the existence of gut-bone axis and suggested that GM and its metabolites are closely involved in the pathogenic process of OA, which might become a potential and promising intervention target. Based on the close coordination of gut-bone axis, this review aims to summarize and discuss the mechanisms of GM and its metabolites influencing OA from the aspects of the intestinal mucosal barrier modulation, intestinal metabolites modulation, immune modulation and strategies for the prevention or treatment of OA based on perspectives of GM and its metabolites, thus providing a profound knowledge and recognition of it.

## Introduction

Osteoarthritis (OA) is a common chronic degenerative disease of articular cartilage in middle-aged and older individuals, mainly manifested as the progressive destruction of articular cartilage, subchondral sclerosis and hyperplasia, periarticular osteophyte formation and synovial lesions, which can result in the joint pain and dysfunction, and even cause the joint deformity or disability (1–3). With the enhancing process of global aging, OA has gradually become a major public health problem worldwide and the main source of social medical expenses for middle-aged and older individuals (4). According to the previous study, by 2020, OA has become the fourth most prevalent disease in the world (5, 6). Meanwhile, due to the high prevalence of OA and the consequent severe consequences for patients, their families and the social medical service system, OA has gradually become a continuous research hotspot in current scientific fields (7, 8).

As a kind of complex chronic degenerative disease, the etiology and pathogenesis of OA are still unclear, and the corresponding treatment or intervention approaches are relatively limited. The current strategies of OA mainly include drug treatment, physical intervention and surgeries. Hence, based on the original cognition of OA (progressive destruction of articular cartilage, subchondral sclerosis and hyperplasia, periarticular osteophyte formation, and synovial lesions), a deeper explanation of the underlying mechanisms of OA is crucial for the development of prevention and treatment strategies. Furthermore, more and more evidence has suggested that gut microbiota (GM) and its metabolites play a vital role in the occurrence and progression of OA (9). As the human intestine contains a large number of microorganisms, GM stores a huge genome, which is about 150 times the number of human genes. Thus, GM is considered to be the second gene bank of human beings, making it an inseparable part of the human body (10–12). Moreover, GM plays a crucial role in the health of host from birth, and the interactions between the intestinal epithelial cells, microorganisms and its metabolites are the key mediators between intestinal epithelium and other cell types (13). This interaction also contributes to maturation of intestinal epithelium, intestinal nervous system, intestinal vascular system and innate immune system (14). In addition, GM influences the basic physiological functions of host (such as digestion and absorption, energy metabolism, and immune defense) through the neuromodulation, immune modulation and endocrine modulation, so as to maintain the homeostasis of internal environment (15).

In recent years, more and more researches have suggested that the dysregulation of GM and its metabolites is not only involved in the pathogenic process of the intestinal diseases (such as inflammatory bowel disease, Crohn’s disease, and colon cancer), but also the imbalance of intestinal homeostasis may induce several extraintestinal immune and metabolic diseases (such as osteoporosis, psoriasis, systemic lupus erythematosus, and OA) (16–21). Regarding this, Sheth et al. (22) showed that approximately 10–40% of patients with inflammatory bowel disease may suffer from at least one extraintestinal lesion, with the most frequent occurrences being musculoskeletal lesions, such as OA and osteoporosis. Schule et al. (23) observed patients in a cohort study of inflammatory bowel disease in the Switzerland, and suggested that 57% of them were combined with osteopenia, 20% were combined with osteoporosis, and part of the remaining patients were combined with OA. Hence, the close correlation between GM and the occurrence and progression of OA can be gradually recognized by the academic circles, which also drives more researchers to explore the advanced nature and profound connotation of OA from the perspective of intestinal microecology. On the basis of close coordination of gut-bone axis (24), this review aims to summarize and discuss the mechanisms of GM and its metabolites influencing OA from the aspects of intestinal mucosal barrier modulation, intestinal metabolites modulation, immune modulation and strategies for the prevention or treatment of OA based on the perspectives of GM and its metabolites, thus providing a profound knowledge and recognition of it.

## The close correlation between osteoarthritis, obesity and gut microbiota

Gut microbiota is the critical factor to activate and maintain intestinal physiological function, and plays an indelible role in maintaining the health and homeostasis of the host. Under normal circumstances, GM maintains a dynamic balance with the host and the external environment (25). Moreover, it is generally recognized that the earliest “immigrants” are the microorganisms in birth canal or the environment that babies come into contact with at birth. At the beginning of the baby’s birth, there is a lot of oxygen in the intestine, mainly due to the colonization of partial bacteria that need oxygen, such as *Escherichia coli* and *Staphylococcus* (26). When they gradually exhausted of oxygen and PH in the intestine decreases, some anaerobic bacteria, such as *Bifidobacteria* and *Bacteroidetes*, begin to colonize. As the baby’s age increases, various bacteria are in the process of mutual influence. Until the baby is 2 years old, a stable GM structure similar to that of adults can be formed (27). After reaching the old age, the decline of GM diversity with the increase of age may be the result of changes in various lifestyles. Bacteria become less diverse, and the number of beneficial microorganisms, such as *Lactobacillus* and *Bifidobacteria*, decreased in number and lost its dominant share (28). These factors also contribute to the imbalance of intestinal microecology and are related to the increased risk of various age-related chronic diseases, such as heart disease, Parkinson’s disease and Alzheimer’s disease (29).

Gut microbiota is symbiotic with host, which affects several vital links, such as nutrition intake, food digestion, systemic metabolism, development of immunity, physiological function maintenance and diseases defense of the human bodies (30, 31). Meanwhile, especially in recent years, more and more human and animal studies have indicated the existence of gut-bone axis and suggested that GM and its metabolites are closely involved in the pathogenic process of OA, which might become a potential and promising intervention target (32, 33). On this basis, Gleason et al. (34) emphasized the role of gut-joint axis in the pathogenic process of OA, and recognized that the gut-joint axis was proposed as an association involving the gastrointestinal microbiome, immune and inflammatory response that it induced, and the overall joint health. Meanwhile, the gut is regarded as an intriguing and novel target for the therapy of OA, and the dietary modification or supplementation with fiber, probiotics, or prebiotics could provide a positive influence on the gut-joint axis (35, 36).

Obesity is a significant risk factor for inducing the OA. In the traditional sense, the excessive joint wear caused by excessive local stress of obese joints is the main cause of OA. However, more and more researches have suggested that excessive joint load cannot fully explain the relationship between obesity and OA (37, 38). Obesity can also increase the prevalence of OA in non-weight-bearing joints, such as hand joints (39, 40). It can be seen that non-mechanical factors are involved in the pathological process of obese OA (41). In this process, the imbalance of GM and its metabolites is a vital trigger factor for the enhancement of inflammation level, and also participates in the occurrence of OA in obese body. Collins et al. (42) showed that the number of intestinal *Lactobacillus* decreased in obese rats, and the abundance of this bacteria was negatively correlated with the levels of inflammatory factors in the blood, synovial fluid and the Mankin score of joints. Meanwhile, the study also indicated that the imbalance of GM and increase of intestinal permeability resulted in enhancement of lipopolysaccharide (LPS) into the blood, and the inflammatory response induced by LPS may be involved in the pathogenesis of OA. Li et al. (43) emphasized the key significance of analyzing the role of nutrients-GM-bacterial metabolites axis in the pathogenesis of OA from different perspectives such as obesity, aging and gender. From the perspective of obesity, the metabolic inflammation, insulin resistance and other diseases caused by the disorder of GM and its metabolites may accelerate the pathological process of OA.

With regard to this, as for the human researches, a well-known perception is that the prevalence and severity of OA in women are much higher than that in men, and the incidence and severity of OA in women after menopause are significantly higher than that before menopause (44). In this process, GM may modulate the immune response by influencing the sex hormones. For example, *Clostridium* can convert glucocorticoids into androgens, and the diversity and richness of bacterial species are positively related to the concentration of serum androgens (45, 46). As GM is involved in the excretion and circulation process of sex hormones, the concept of “microgenderome” indicating the role of sex hormone on the GM has been gradually proposed (47). Meanwhile, GM also presents a dynamic change process in body, and the types of GM and beneficial bacteria enhance from the infants. When body begins to decline with age, the abundance and diversity of GM also decreases, and harmful bacteria increases (48). A population-based study conducted by the Xiangya OA team (49) included a total of 1388 middle-aged and older individuals, and revealed that the alterations in the composition of GM were observed among study participants who had symptomatic hand OA, and a low relative abundance of *Roseburia* but high relative abundance of *Bilophila* and *Desulfovibrio* at genus level were associated with prevalent symptomatic hand OA. In a population-based study, Rushing et al. (50) revealed that the participants with obesity and knee plus hand OA had distinct fecal metabolomes characterized by the enhanced products of proteolysis, perturbations in leukotriene metabolism, and alterations in the microbial metabolites compared with healthy controls. Chen et al. (51) analyzed GM of 57 patients with OA and a corresponding number of healthy controls, and observed that the abundance and diversity of GM in the patients with OA reduced significantly, mainly manifested in the decrease of *Bifidobacterium longum* and *Faecalibacterium*, while the level of *Clostridium* enhanced. A prospective study led by Wang et al. (52) included 182 stool samples from overweight OA patients (86 cases) and overweight normal subjects (96 cases) and found that the diversity and richness of GM in the overweight OA patients decreased. Accordingly, there were 9 branches and 87 genera with significant difference between the overweight OA patients and overweight normal subjects. Lee et al. (53) reported that the ratio of *Bacteroidetes* and *Firmicutes* can be regarded as one of potential indicators to predict the occurrence and severity of OA and rheumatoid arthritis, but whether other specific species and genera can also play a predictive role needs to be further explored in the future researches. The literatures regarding the GM and OA in population-based studies are summarized in Table 1 (54–56). Hence, it could be acknowledged that more and more evidence indicate that GM participates in the pathological modulation of OA (57), and exhibits differences in composition, abundance and diversity at the different levels. Besides, the gender, age, dietary habits, obesity, lifestyle and other factors might also be the risk factors linking between the GM and OA.

**TABLE 1:** The literatures regarding the GM and OA in the population-based studies.

Population	Study size, *n*	Intervention mode	Outcomes	References
Middle-aged and older individuals with OA, and the controls.	1388	GM was analyzed using 16S ribosomal RNA gene sequencing in stool samples.	Higher relative abundance of genera *Bilophila* and *Desulfovibrio* as well as lower relative abundance of the genus *Roseburia* was associated with symptomatic hand OA	Wei et al. (49)
Participants with obesity and knee plus hand OA, and the controls.	92	Fecal metabolomes were analyzed by a UHPLC/Q Exactive HFx mass spectrometer, and microbiome composition was determined in fecal samples by 16S ribosomal RNA amplicon sequencing.	Adults with obesity and knee plus hand OA have distinct fecal metabolomes characterized by increased products of proteolysis, perturbations in leukotriene metabolism, and changes in microbial metabolites compared with controls.	Rushing et al. (50)
OA patients and the healthy controls	114	Metagenomic shotgun sequencing was conducted.	A significant reduction in the richness and diversity of GM were observed in OA patients. *Bifidobacterium longum* and *Faecalibacterium prausnitzii* were decreased while *Clostridium* spp. was increased in OA group. The functional modules, particularly the energetic metabolism and acetate production were also decreased in OA patients.	Chen et al. (51)
Overweight OA patients and overweight normal people.	182	16S ribosomal RNA gene sequencing for V3 and V4 regions on the Illumina MiSeq platform was conducted.	Both the diversity and richness of GM decreased in overweight OA patients, and 9 phyla and 87 genera had significantly difference between overweight OA patients and overweight normal people.	Wang et al. (52)
RA patients and OA patients.	18	Stool samples were collected and DNA was extracted, and the GM was assessed using 16S rRNA gene amplicon sequencing.	The microbial properties of the gut differed between RA and OA patients, and the RA dysbiosis revealed results similar to those of other autoimmune diseases.	Lee et al. (53)
KBD patients and normal controls.	67	Fecal and serum samples were used to characterize the GM using 16S rDNA gene and metabolomic sequencing via liquid chromatography-mass spectrometry.	The KBD group was characterized by elevated levels of *Fusobacteria* and *Bacteroidetes*, and a total of 56 genera were identified to be significantly differentially abundant between the two groups. The genera *Alloprevotella*, *Robinsoniella*, *Megamonas*, and *Escherichia_Shigella* were more abundant in KBD group.	Wang et al. (54)
Knee OA patients and normal individuals with or without vitamin D deficiency.	24	The fecal samples of all the participants were taken for DNA extraction. The V3–V4 region of 16s rRNA was amplified, and the library was prepared and sequenced on the Illumina Miseq platform.	*Parabacteroides*, *Butyricimonas*, *Pseudobutyrivibrio*, *Odoribacter*, and *Gordonibacter* are the predominant bacteria in vitamin D deficient patients with or without Knee OA, and these results indicate an association between GM, vitamin D and Knee OA.	Ramasamy et al. (55)
OA and KBD patients.	64	Metagenomic sequencing of fecal samples from OA and KBD subjects was performed.	*Streptococcus_parasanguinis*, and *Streptococcus_salivarius* were significantly increased in abundance in the OA group compared to those in the KBD group, and the species *Prevotella_copri*, *Prevotella_*sp*._CAG:386*, and *Prevotella_stercorea* were significantly decreased in abundance in OA group compared to those in KBD group by using metagenomic sequencing.	Ning et al. (56)
OA patients had hand and knee OA and healthy controls.	92	Compositional analysis of stool samples was carried out by 16S ribosomal RNA amplicon sequencing.	The lack of differences in the GM, but increased serum LPS levels, suggest the possibility that increased intestinal permeability allowing for greater absorption of LPS, rather than a dysbiotic microbiota, may contribute to the development of OA associated with obesity.	Loeser et al. (57)

GM, gut microbiota; OA, osteoarthritis; RA, rheumatoid arthritis; KBD, Kashin–Beck disease; LPS, lipopolysaccharide.

In addition, as for the animal researches, Guan et al. (58) indicated that in the mice with injury-induced OA, the imbalance of GM induced by antibiotics could reduce the serum LPS level and inflammatory response, and further improve the OA after joint injury. Collins et al. (42) explored the link between GM, systemic LPS levels, serum and local inflammatory profiles and OA in a high fat/high sucrose diet-induced obese rat model, and discovered the close interaction relationship between GM and adiposity-derived inflammation and metabolic OA. Ulici et al. (59) observed that compared with specific pathogen free (SPF) mice with injury-induced OA, the germ-free (GF) mice were equipped with better synovial score and articular cartilage structure, indicating the factors related to GM promoted the development of OA after joint injury. Moreover, Hahn et al. (60) compared the early responses to injury-induced OA in the conventional and GF mice, and revealed that the differences in response to injury in GF compared to conventional mice were related to metabolic pathways that modulated the inflammation associated with the innate immune system. The literatures regarding the GM and OA in animal studies are summarized in Table 2 (61–64). Hence, on the basis of these animal studies, the summarized evidence gradually exhibited a direct gut-bone communication that set the stage for novel approaches of exploring OA pathogenesis and potentially new OA therapeutics.

**TABLE 2:** The literatures regarding the GM and OA in the animal researches.

Animal models	Study size, *n*	Modeling methods	Outcomes	References
8-week-old C57BL/6N mice.	54	Destabilized medial meniscus	The imbalance of GM induced by antibiotics could reduce the serum lipopolysaccharide level and inflammatory response, and further improve the OA after joint injury.	Guan et al. (58)
8–12 weeks old SD rats.	32	Diet-induced obese model.	Increased OA in diet-induced obese animals is associated with greater body fat, not body mass. The link between GM and adiposity-derived inflammation and metabolic OA warrants further investigation.	Collins et al. (42)
8-week-old GF C57BL/6J male mice.	20	Destabilized medial meniscus.	Compared with specific pathogen free mice with injury-induced OA, the GF mice were equipped with better synovial score and articular cartilage structure, indicating the factors related to GM promoted the development of OA after joint injury.	Ulici et al. (59)
20-week-old GF and conventional C57BL/6 mice.	18	Non-invasive anterior cruciate ligament rupture.	Conventional mice had greater variability in their metabolic response to injury, and a more distinct joint metabolome compared to their corresponding controls. The differences in response to injury in GF compared to conventional mice were linked to mouse metabolic pathways that regulate inflammation linked with innate immune system.	Hahn et al. (60)
8-week-old GF C57BL/6J mice	42	Meniscal/ligamentous injury.	Fecal microbiota transplantation from the metabolically compromised human donors is able to accelerate the process of OA in mice, and the alterations of *Fusobacterium*, *Faecalibaterium*, and *Ruminococcaceae* exhibited a role of these particular microbes in exacerbating OA.	Huang et al. (152)
8-week-old male Wistar rats.	27	Monosodium iodoacetate-induced Knee OA model.	Moxibustion treatment led to significant improvements in GM and inflammatory factors of rats with Knee OA. Moxibustion treatment of 4 and 6 weeks led to better outcomes than the 2-week course. Moxibustion for 4 and 6 weeks can regulate GM dysfunction with increased probiotics and reduced pathogenic bacteria, reduce pro-inflammatory factors and increase anti-inflammatory factors. No significant differences were seen between the effects of moxibustion for 4 weeks and 6 weeks.	Jia et al. (61)
12-week-old GF C57BL/6J male mice.	54	High-fat diet.	High-fat diet decreased gut microbial diversity. Increase in endotoxin-producing bacteria, decrease in gut barrier-protecting bacteria, high LPS levels in the blood and synovial fluid, high TLR4 and MMP-13 expression levels, and severe cartilage degeneration were observed. Exercise caused high gut microbial diversity. The GM was reshaped, LPS levels in the blood and synovial fluid and TLR4 and MMP-13 expression levels were low, and cartilage degeneration was ameliorated.	Li et al. (62)
4-week-old male C57BL/6J mice.	12	Non-invasive ACL rupture using a single dynamic tibial compressive overload using an electromagnetic material testing system.	A reduced state of inflammation at the time of injury and a lower expression of Wnt signaling modulatory protein, Rspo1, caused by antibiotic treatment can slow down or improve PTOA outcomes.	Mendez et al. (63)
12-week-old SD male rats.	48	High-fat/high-sucrose diet-induced obese model.	Prebiotic fiber supplementation, aerobic exercise, and the combination of the two interventions completely prevented knee joint damage that is otherwise observed in this rat model of obesity. Prevention of knee damage was associated with a normalization of insulin resistance, leptin levels, dyslipidemia, gut microbiota, and endotoxemia in the high-fat/high-sucrose –fed rats.	Rios et al. (64)

GM, gut microbiota; OA, osteoarthritis; GF, germ-free; LPS, lipopolysaccharide; TLR4, toll-like receptors-4; MMP-13, matrix metalloprotinase-13; Rspo1, R-spondin-1; PTOA, post-traumatic osteoarthritis.

## The involvement of intestinal mucosal barrier modulation

The intestinal barrier is composed of mechanical barrier, chemical barrier, immune barrier and biological barrier. The mechanical barrier is mainly composed of intestinal mucosal epithelial cells, intercellular tight junctions and bacterial membranes, which could effectively prevent harmful tissues, such as bacteria and endotoxin, from entering the blood through the intestinal mucosa (65, 66). The chemical barrier consists of mucus secreted by epithelium, digestive juice and antibacterial materials produced by parasitic bacteria (67). The immune barrier is composed of intestinal mucosal lymphoid tissues (mesenteric lymph nodes and intraepithelial lymphocytes), which could generate local mucosal immune response and specifically secrete secretory immunoglobulin A (sIgA) to prevent the damage of pathogenic antigens (68). The biological barrier is composed of the microspatial structure formed between the normal parasitic flora of intestinal tract and host intestinal tissue (69). Hernandez et al. (70) showed that GM and its metabolites can cause the abnormal intestinal mucosal barrier function by altering intestinal nutrient absorption, inducing the changes in intestinal immune function and abnormal intestinal endothelial transport, thus inducing the occurrence and progression of extraintestinal diseases, such as OA and osteoporosis.

The permeability of intestinal mucosa generally allows substances with a molecular weight >150 KD to readily pass through the intestinal epithelium and then enter into the circulation (71). Therein, the permeation modes of intestinal epithelial permeability include transepithelial pathway and paracellular pathway. The transepithelial pathway is that nutrients are absorbed from the intestinal cavity through the intestinal mucosal epithelium and enter into the circulation, while selectively restricting the passage of harmful substances, such as endotoxin and inflammatory factors (72). The paracellular pathway is the passive diffusion of the intestinal molecules through the epithelial space, which is controlled by the tight junction between the epithelial cells and modulates the passage of intestinal microflora products and other macromolecules (73). The changes of intestinal mucosal permeability are related to various factors, including exogenous factors, nutritional factors, several kinds of cytokines, and effects of immune cells. The alterations of GM can affect the absorption of vitamin B (Vit B), vitamin C (Vit C), vitamin D (Vit D), vitamin K (Vit K) and tetrahydrofolate, metabolism of carbohydrate and fats, the synthesis of short chain fatty acids (SCFAs), thus influencing the stability, growth and progression of intestinal mucosal cells. During this process, the primary focus of metabolites mainly includes SCFAs, 5-hydroxytryptamine (5-HT), tryptophan-related metabolites, and so on.

Importantly, SCFAs are the main products of GM fermenting resistant starch and non-starch polysaccharides (the main components of dietary fiber) (74). When GM is disturbed, the types and contents of SCFAs in the intestine could be significantly altered. Therein, the acetic acid, propionic acid and butyric acid have been generally verified by several researches to be involved in the bone metabolism (75, 76). Collins et al. (42) revealed that high-fat diet (HFD) can significantly reduce the amount of *Lactobacillus* and the synthesis of SCFAs in the intestine, thereby decreasing the energy supply of intestinal cells, and the bacterial abundance was closely associated with the levels of inflammatory factors in the blood and synovial fluid of the rat models with OA. As one of the significant components of intestinal mucosal barrier, the expression of the tight junction protein, zonula occludens 1 (ZO-1), is prone to be reduced by HFD, and the intestinal permeability also alters, resulting in a large amount of LPS entering systemic circulation (77). Meanwhile, HFD also induces large amounts of saturated fat into the intestine, enhances the number of activated macrophages, thus releasing additional pro-inflammatory mediators and adipokines, directly amplifying the inflammatory response and aggravating the clinical outcome of OA (78). Based on this, LPS widely exists in the outer membrane of intestinal Gram-negative bacteria that can induce inflammatory response, and its excessive translocation into the body circulation might be one of the predisposing factors of OA. LPS can also activate innate immune system cells, such as macrophages and neutrophils, and synthesize the proinflammatory cytokines, including interleukin (IL)-1β, tumor necrosis factor (TNF)-α and so on, resulting in the secondary inflammation in various joints throughout the body (57).

In addition to this, 5-HT could disrupt the dynamic balance between bone formation and bone resorption, thereby influencing the maintenance of bone mass. GM plays an important role in the production of 5-HT, and previous studies have also indicated that GM can stimulate the chromaffin cells to produce 5-HT, and microorganisms can also regulate gastrointestinal motility via the neural secretion mechanism activated by toll like receptors (TLRs) (79, 80). Besides, 5-HT has dual effects on bone metabolism. As a hormone, intestine-derived 5-HT can inhibit bone formation, and as a neurotransmitter, the brain-derived 5-HT can promote bone formation through a series of pathways (81). Tryptophan is a kind of aromatic amino acid, which can act as the biosynthetic precursor of certain microorganisms and host metabolites, thereby modulating the physiological functions of the body (82). Tryptophan is mainly metabolized in intestine through the following three pathways: (1) enterochromaffin cells synthesize most of the 5-HT in the circulation via tryptophan hydroxylase 1 (TPH 1); (2) partial metabolites can induce the indoleamine 2,3-dioxygenase 1 (IDO1) and promotes the metabolism of tryptophan to kynurenine; (3) under the action of GM, tryptophan is directly converted into the indoles and acts as an aromatic hydrocarbon receptor ligand (83).

In addition, GM could also modulate and inhibit the dietary inflammatory response produced in the daily intake of foods, which also play a critical role as antigens in the immune system (84). As a kind of transmembrane transporter, the microbe-associated molecular patterns (MAMPs) could affect the physiological activities of distant organs and tissues after entering the systemic circulation. Kim et al. (85) reported that MAMPs could directly influence the bone remodeling by stimulating innate immune receptors on osteocytes, including modulating the balance between osteoblasts and osteoclasts. When the MAMPs are translocated, the living bacteria could pass through the intestinal mucosal barrier, the translocated bacteria are usually quickly cleared by immune cells, and the uncleared bacteria enter with MAMPs and stay in the intestinal mucosal barrier. After inducing the death of immune cells within the barrier, a small amount of MAMPs may be released into systemic circulation. Furthermore, as MAMPs circulate to distant organs and tissues, such as several joints of limbs, MAMPs can activate the immune response and produce the local inflammation (86). Partial bacteria that pass through the intestinal mucosal barrier can penetrate the natural cells and survive, thus avoiding the impact of inflammatory response (87). Generally, it can be recognized that the changes of intestinal mucosal permeability and the transport of GM-related metabolites across the intestinal mucosal barrier could influence the occurrence and progression of OA to a certain extent.

## The involvement of immune modulation

There is a close relationship between GM and intestinal mucosal immune system, and the degree of change in intestinal microecology determines the stability of intestinal mucosal immune system and systemic immune system. The direct contact between GM, its metabolites and immune cells stimulates the immune response of intestinal mucosal barrier (88–91). The immune cells (such as T cells and dendritic cells) can interact with GM, and migrate to lymph nodes to activate the pro-inflammatory or anti-inflammatory responses. Meanwhile, these cells are also able to release soluble pro-inflammatory or anti-inflammatory mediators or cytokines into the systemic circulation and participate in the modulation of systemic bone remodeling (92, 93). In addition to this, TLR also serve as a bridge between the intestinal mucosal barrier, GM and the innate immune system, and play a crucial role in the formation of GM. The intestine is rich in dietary and microbial antigens, and the human immune system needs to tolerate these antigens to create conditions for survival of GM. TLR is one of the significant immunosensors, which maintains the balance of GM in the whole body (94). TLR exists in a variety of innate immune system cells, such as macrophages, dendritic cells (DCs) and intestinal epithelial cells, which could recognize the pathogenic microorganisms and induce the production of antibacterial products, so as to modulate immune response (95–97). There is also a critical link between the innate immune response and cartilage damage, which affects the development of OA. Articular cartilage damage caused by several factors, such as aging and obesity, might trigger the release of autoantigens and damage-associated molecular patterns (DAMPs), thereby activating the innate immune response (the activation of immune cells, activation of complement system and release of anti-cartilage components in the immune complexes), resulting in the generation of sterile inflammation and further damage to the cartilage, and forming a vicious circle (98, 99).

As a widely reported immunomodulator, GM mainly stimulates the host intestinal wall to collect the lymph nodes through the bacteria itself or cell wall components, and generates immune response to bacteria (100, 101). During this process, the lymphocyte activation transmits the immune response to the entire intestinal mucosa, and then forms secretory IgA globulin to cover the mucosal surface. Regarding this, IgA globulin is the largest amount of immunoglobulin secreted by the body, which could prevent the adhesion of pathogenic microorganisms in intestine from attaching to mucosal surface, neutralize bacterial toxins, and play a synergistic bactericidal role with complement and lysozyme (102, 103). Moreover, GM could directly modulate the immune cells, such as *Prevotella copri* can promote the differentiation of regulatory T (Tregs) cells and Th17 cells in the intestine, and Tregs cells induce the production of retinoic acid-related orphan receptor γt (RORγt) to further promote the differentiation of Th17 cells (80, 104). On the basis of this, GM could modulate the type II hypersensitivity through Th17 cells and Tregs cells, so as to balance the immune response on the surface of intestinal mucosa (105). Moreover, Ivanov et al. (106) indicated that the segmented filamentous bacteria (SFB) can induce the differentiation of Th17 cells, and *Bacteroides fragilis* can also induce the differentiation of Th1 cells and Treg cells. Atarashi et al. (107) proposed that when the GM subsets (such as SFB, *Citrobacter*, *Escherichia coli O157* and certain extracellular pathogens) colonized and adhered to the intestinal epithelial cells, the intestinal Th17 cells were then induced and aggregated. In a recent study involving the oral-GM axis, du Teil Espina et al. (108) noticed that one of the causes of the rheumatoid arthritis was the alterations of oral bacteria, and the oral diseases (such as periodontitis) could result in the GM disorder, and its diversity and abundance may also be reduced, while the formation of neutrophils and biofilm plays an indelible role in this process. Besides, intestinal citrullinated proteins mediated by oral pathogens (*Porphyromonas gingivalis* and *Actinobacillus*) can promote the production of anti-citrullinated protein antibodies (109, 110). In this process, the overgrown GM might strengthen the response of Th1 cells and further inhibit the activation of Tregs cells by inhibiting the growth of *B. fragilis*, leading to immune imbalance and the occurrence and progression of rheumatoid arthritis (111). Hence, it is recognized that the in-depth exploration of immune modulation caused by the alterations and dysregulation of GM provides a promising intervention target for the prevention and treatment of OA in the future.

## The strategies for the prevention or treatment of osteoarthritis based on the perspectives of gut microbiota and its metabolites

During the process of ongoing researches, the researchers unexpectedly observed that in the treatment of the patients with intestinal or extraintestinal diseases caused by intestinal microecological disorders, partial patients with OA exhibited joint pain relief and improved exercise ability with the advancement of treatment process (112), which provided a novel perspective and ideas for the basic researches and clinical treatment of OA. With regard to this, the intestine is the main site of nutrient absorption in human bodies, and the daily dietary intake and supplementation of auxiliary substances has a profound and dynamic impact on the composition and function of GM (113–115). Diets play a significant role in shaping the structure and function of GM, and the GM triggers numerous signal molecules with host receptors by metabolizing food-derived SCFAs and signaling molecules, such as trimethylamine oxide (TMAO), directly or indirectly influencing the distant organs (bone, kidney, brain, skin, and so on) (116, 117). The beneficial metabolites of specific foods contribute to enriching the intestinal beneficial bacteria to play a positive role in modulating the homeostasis of body (118, 119). Hence, the structure and function of GM can also be modulated by supplementing probiotics and prebiotics. Probiotics cannot only restore the bone loss caused by hypogonadism or ovariectomy, but also work to improve the inflammatory bone-related diseases (120).

Diet is closely related to the pathogenesis of OA, and the selenium, magnesium and vitamins play a significant role in the pathogenesis of OA (121–123). Moreover, high-glucose diets and HFDs are also closely associated with the OA. The glucose plays an important role in the growth and development of articular cartilage, and the disorder of glucose metabolism may aggravate the progression of OA (124). More and more recent studies have revealed that GM also has a regulatory effect on the blood glucose, and the number of *Akkermansia muciniphila* (*A. muciniphila*) in the type II diabetic and obese mice is significantly reduced. However, when the mice are fed with polyphenol-rich cranberry extract, the number of *A. muciniphila* increases significantly, and the glucose tolerance and insulin sensitivity are improved accordingly (125, 126). Similarly, the excessive intake of HFD might also result in the alterations of GM, and Nguyen et al. (127) also reported that the supplementation of *Lactobacillus plantarum* PH04 could effectively reduce the cholesterol and triglyceride levels of hypercholesterolemia mice. Ramasamy et al. (55) suggested in a pilot study that *Parabacteroides*, *Butyricimonas*, *Pseudobutyrivibrio*, *Odoribacter*, and *Gordonibacter* are predominant bacteria in Vit D deficient patients with or without Knee OA, and these results noticed a non-negligible link between GM, Vit D and Knee OA. Thus, it is recognized that the diets-GM axis is closely associated with the glucose and lipid metabolism of the body, and altering the intake of dietary nutrients might be an effective approach to alleviate OA.

In clinical practice, the proper supplementation of microecological regulators tends to be used to correct the intestinal microecological disorders and maintain homeostasis modulation. Currently, the probiotics and prebiotics are widely used in clinic and daily life (128). On one hand, the probiotics are a kind of microorganisms that have beneficial effects on the host after sufficient intake, and play an active role mainly by altering the composition and structure of GM and metabolic activities. By supplementing sufficient probiotics, supporting the beneficial bacteria in the body could improve the resistance to pathogenic bacteria, modulate the function of intestinal mucosal barrier, optimize the intestinal environment, and maintain the immune balance of body (129–131). Tyagi et al. (132) observed that the supplementation of *Lactobacillus rhamnosus* GG is able to increase the butyric acid-producing bacteria in the intestine of mice, promote the production of butyric acid, and then improve the expression of Wnt10b and increase the bone mass through the Tregs cells, so as to participate in the modulation of bone metabolism. Pan et al. (133) revealed that *Lactobacillus casei* can correct the imbalance of GM, restore the abundance of *Lactobacillus gasseri*, *Lactobacillus reuteri*, and *Lactobacillus vaginalis* to normal levels, and enhance the abundance of *Lactobacillus acidophilus*, thereby protecting the bones from destruction in adjuvant-induced arthritis (AIA) rats. Henrotin et al. (134) administered orally a freeze-dried inactivated culture of the *Bifidobacterium longum* CBi0703 in a spontaneous model of OA in guinea pigs, and observed that the cartilage structural injury and the degradation of type II collagen caused by OA were significantly reduced, which has potential to prevent and treat OA. Sim et al. (135) also regularly fed the tyndallized *Clostridium butyricum* to the rats to treat or alleviate OA, and experimental results revealed that the levels of inflammatory indicators and bone metabolism markers in the serum were significantly reduced, while the concentrations of interferon-γ (IFN-γ) and glycosaminoglycans were significantly enhanced. As a consequence, the application of tyndallized *C. butyricum* also effectively protected the cartilage and synovium of knee joint, and reduce the formation of fibrous tissue. In addition to this, by comparing applications of probiotic containing capsules, including the *Lactobacillus rhamnosus*, *Saccharomyces cerevisiae* (*boulardii*), and *Bifidobacterium animalis* ssp. *lactis*, and placebo capsules without probiotics to the participants with OA, Taye et al. (136) found that although the reduction of pain scores related to probiotic intervention was relatively small, the application of probiotics had a clinical practical significance for the pain management of OA patients. Moreover, as a newly founded bacterium from human breast milk, *TCI633* (*Streptococcus thermophilus*) is able to produce hyaluronate in gastrointestinal tract. Lyu et al. (137) recruited 80 patients with knee OA and conducted a double-blind controlled study with *TCI633* and placebo, and the results suggested that TCI633 might be able to delay the progression of knee OA.

On the other hand, the prebiotics are a kind of indigestible food components that can play a beneficial role in the host by altering the composition of GM and the activity of partial GM, or being fermented by partial GM, and can also stimulate and activate intestinal probiotics (138, 139). Prebiotics are considered as the auxiliary substrates for the bacterial activation and growth promotion, which can resist the action of hydrolytic enzymes and a variety of proteases, and are converted into SCFAs after being fermented by GM (140). Schott et al. (141) observed that *Bifidobacterium* in the intestine of obese rats were significantly reduced, and the alterations of GM resulted in the accumulation of downstream systemic inflammatory signals along with macrophages to the synovium of joints, which accelerated the progression of OA. However, the restoration of GM by the addition of *Fructooligosaccharides* decreased the systemic levels of inflammation and alleviated the OA-related symptoms. Therein, the relevant mechanism may be that the supplementation of *Fructooligosaccharides* can increase the level of *Lactobacillus*, thus maintaining the intestinal mucosal barrier, preventing bacterial endotoxin or LPS translocation, and inhibiting the proliferation of *Enterobacteriaceae*. Tanabe et al. (142) further explored the effects of prebiotics on bone metabolism in the aging mice, and the results indicated that *Fructooligosaccharides* and *Glucomannan* could inhibit the bone absorption and improve the bone metabolism in aging mice, and further obtain the aim of increasing femoral calcium contents mainly by enhancing the level of *Lactobacillus*, *Bacteroides* and *Clostridium*. Furthermore, *Fructooligosaccharides* are not easy to be absorbed by the human bodies, and can resist the digestive reaction of intestine, so that *Fructooligosaccharides* could reach the colon smoothly after intaking it, become the growth matrix to promote GM optimization, and indirectly have corresponding effects on the human bodies (143, 144). Meanwhile, Wu et al. (145) also indicated that the direct oral administration of *Fructooligosaccharides* could alleviate the inflammatory response of mouse pups without altering the type, quantity and abundance of GM in mice. This result suggested that the prebiotics might also play a direct role in immune modulation without the intervention of GM. Hence, especially for the obesity-related OA (mainly manifested as an inflammatory reaction process), which is related to the obesity-related GM dysregulation, and could restore a healthy microbial community via the use of indigestible prebiotic fiber *Fructooligosaccharides*. *Fructooligosaccharides* can induce the alterations in the abundance of key microorganisms in obese individuals, decrease the response characteristics of colon macrophages, reduce the systemic and joint inflammation, protect the articular cartilage, and further alleviate the progression of OA (146, 147). Thus, more and more evidence has revealed that the supplementation of probiotics and prebiotics could reduce the expression of pro-inflammatory cytokines and cartilage-degrading factors in several joint tissues, enhance the production of anti-inflammatory factors and cartilage matrix synthesis cytokines, so as to inhibit combined inflammation and cartilage destruction, and alleviate the development process of OA.

Furthermore, as a research focus in recent years, fecal microbiota transplantation (FMT) often refers to the transplantation of bacteria in the feces of donor to the recipient by intragastric or oral administration, thereby altering the composition, structure and abundance of GM in the recipients (148, 149). During this process, FMT can reshape the intestinal microenvironment, improve the inflammation, immunity and metabolism of the recipient’s gut, and provide a new treatment concept and method for a variety of intestinal and parenteral diseases (150). Huang et al. (151) suggested that FMT from the metabolically compromised human donors was able to accelerate the process of OA in mice, and the alterations of *Fusobacterium*, *Faecalibaterium* and *Ruminococcaceae* exhibited a vital role of these particular microbes in exacerbating the OA. Additionally, glucosamine sulfate and chondroitin sulfate are the proteoglycan-based nutraceuticals. Theoretically, taking it is able to stimulate chondrocytes to produce new collagen and proteoglycans, thus helping body repair cartilage damaged by OA (152, 153). However, partial studies have shown that these compounds can usually reduce pain caused by OA and repair articular cartilage within a few weeks to months after starting treatment (154). Therefore, although more and more OA patients are currently using it worldwide, larger randomized controlled clinical trials are still needed to verify the safety and efficacy of glucosamine sulfate and chondroitin sulfate on OA. Furthermore, the non-steroidal anti-inflammatory drugs (NSAIDs) are commonly used as a treatment method for OA, and several previous reports also revealed the role of NSAIDs in the regulation of OA from the perspective of GM. Boer et al. (155) suggested that the administration of NSAIDs can affect the diversity and abundance of GM, and there is a certain correlation between the abundance of specific bacterial groups and the pain degree of knee OA. Vitetta et al. (156) reported that the pharmaceuticals to treat OA-related pain (such as NSAIDs) may disrupt intestinal barrier integrity and induce intestinal inflammation. In general, for patients who often need to take NSAIDs to alleviate the OA-related pain, the GM disorders characterized by abnormal proliferation of Gram-negative bacteria can induce abnormal intestinal mucosal immune response, which can cause intestinal inflammation and intestinal injury, while probiotics and rifaximin can significantly alleviate intestinal injury induced by NSAIDs (157, 158).

## Conclusion and perspectives

**FIGURE 1:**
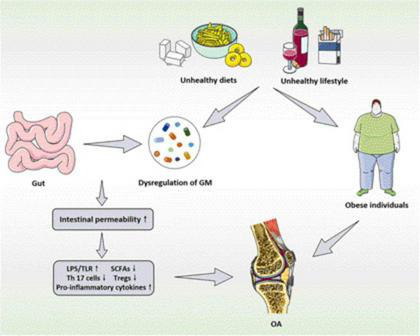
The functional relationship and mechanisms between GM and its metabolites and OA. GM, gut microbiota; OA, osteoarthritis; LPS, lipopolysaccharide; TLR, toll-like receptors; SCFAs, short chain fatty acids; Tregs, regulatory T.

To sum up, with the in-depth researches of the pathogenesis of OA, more and more evidence indicates that there is a close relationship between OA and GM. Although the specific mechanisms of the relationship between intestinal microecology and OA is still unclear and under intensive exploration, it is foreseeable that the characteristics of GM and its metabolites can be applied to diagnose and evaluate the OA or other bone-related diseases in the future. The functional relationship and mechanisms between GM and its metabolites and OA are summarized in Figure 1. Meanwhile, improving the intestinal microecology can also be developed as a potential therapeutic approach to prevent and intervene the development process of OA. Nevertheless, in the process of improving the understanding of GM and bone-related diseases represented by OA, there are still partial unavoidable challenges need to be emphasized, including (1) due to the relatively slow rate of bone alterations, it is difficult to experimentally observe the continuous alterations of GM through the current experiments. Furthermore, among the existing research methods, a common method of utilizing the GM is to transfer the microbiota from the donor to GF animals. When exposed to a new host environment, the structure of transferred microbiota might also alter over time; (2) the preclinical studies related to OA have focused on adult skeletal phenotypes, which require the application of older animals. The adult phenotype may be sensitive to the time of microbial exposure, so it is difficult to assess the GM changes related to mature bones; (3) there are still few methods to effectively utilize GM and its metabolites to obtain the targeted therapeutic effects, which need to be further explored. Hence, in the course of future exploration, repetitive animal studies and clinical trials should be conducted in response to the above challenges to examine the growth, quality, structure, strength and bone healing of the experimental animals and human bones in the presence of GM dysregulation, so that the GM and its metabolites might become a promising target for the prevention and treatment of bone-related diseases represented by OA.

## Author contributions

LL, FT, and RX developed the idea for this manuscript. LL, FT, and WX developed the search strategy and performed the review and data extraction. LL and FT wrote the manuscript. All authors edited and agreed the final version.
